# Efficacy and safety of stiripentol in the prevention and cessation of status epilepticus: A systematic review

**DOI:** 10.1002/epi4.13036

**Published:** 2024-10-03

**Authors:** Nicola Specchio, Stéphane Auvin, Adam Strzelczyk, Francesco Brigo, Vicente Villanueva, Eugen Trinka

**Affiliations:** ^1^ Neurology, Epilepsy and Movement Disorders Unit, Bambino Gesù Children's Hospital IRCCS, Member of ERN EpiCARE Rome Italy; ^2^ Université Paris Cité, INSERM NeuroDiderot Paris France; ^3^ APHP, Robert Debré University Hospital, Pediatric Neurology Department CRMR Epilepsies Rares, Member of ERN EpiCARE Paris France; ^4^ Institut Universitaire de France (IUF) Paris France; ^5^ Epilepsy Center Frankfurt Rhine‐Main, Department of Neurology University Hospital Frankfurt, Goethe‐University Frankfurt Frankfurt am Main Germany; ^6^ Department of Neurology Hospital of Merano (SABES‐ASDAA) Merano Italy; ^7^ Refractory Epilepsy Unit, Neurology Service Hospital Universitario y Politécnico La Fe, Member of ERN EpiCARE Valencia Spain; ^8^ Department of Neurology, Christian‐Doppler University Hospital, Centre for Cognitive Neuroscience Paracelsus Medical University, Member of ERN EpiCARE Salzburg Austria; ^9^ Centre for Cognitive Neuroscience, Christian‐Doppler University Hospital, Neuroscience Institute Paracelsus Medical University Salzburg Austria; ^10^ Institute of Public Health, Medical Decision‐Making and HTA, UMIT – Private University for Health Sciences, Medical Informatics and Technology Hall in Tyrol Austria

**Keywords:** acute seizures, antiseizure medication, developmental and epileptic encephalopathy, Dravet syndrome, hospitalization, morbidity

## Abstract

Status epilepticus (SE) is a life‐threatening emergency with high morbidity and mortality. In people with epilepsy, the management of SE is focused on early medical treatment. Stiripentol is a third‐generation antiseizure medication (ASM) approved for refractory generalized tonic–clonic seizures in Dravet syndrome. The aim of this systematic review was to evaluate the effectiveness and safety of stiripentol in reducing the incidence of SE in patients with Dravet syndrome or any epilepsy characterized by recurrent SE. The PubMed and Cochrane databases were systematically searched, and gray literature was hand‐searched. Search results were screened by title and abstract; studies with data on the effect of stiripentol on SE outcomes, including the cessation of SE, reduction in number of SE episodes, or reduction in hospitalizations, were included. Of 66 records identified, 17 studies were eligible for inclusion, of which 15 were human studies (*n* = 474; aged 1.1–78 years), and two were animal experiments. Results of retrospective or prospective observational studies showed that stiripentol as add‐on therapy to ASMs such as clobazam or valproate reduced the incidence of SE in patients with Dravet syndrome or other developmental and epileptic encephalopathies (DEEs). A mean of 68% of patients (range 41%–100%) had a ≥50% reduction in SE episodes from baseline, and 26%–100% of patients (mean 77%) became SE‐free after stiripentol initiation. Moreover, this review found stiripentol, used as acute treatment, may also be effective for the cessation of super‐refractory SE, but data are limited to three retrospective case series. Stiripentol was generally well‐tolerated. In conclusion, stiripentol reduces the incidence of SE episodes in patients with Dravet syndrome and potentially other DEEs, and it promotes cessation of super‐refractory SE in patients with and without a history of seizures.

**Plain Language Summary:**

Status epilepticus (SE) is a life‐threatening, long‐lasting seizure occurring in patients with/without epilepsy. This article analyzed 15 published studies that investigated the effects and safety of the anti‐seizure medication stiripentol for preventing SE in epilepsy patients (prevention) or stopping an SE episode (cessation), and two animal studies that investigated how stiripentol works. In epilepsy patients, stiripentol halved the number of SE episodes in 41–100% of patients, 26–100% of patients became SE‐free, and stiripentol was considered to be well tolerated. In patients with/without epilepsy, stiripentol may stop the SE episode after other drugs like anesthetics have not worked.

**Key points**

- Stiripentol prevents status epilepticus (SE) in patients with Dravet syndrome or other developmental and epileptic encephalopathies.
- This systematic literature review shows that almost 80% of patients suffering from SE become SE‐free after stiripentol initiation.
- Stiripentol may also be effective for the termination of active super‐refractory SE in patients with and without a history of seizures.
- Stiripentol is generally well‐tolerated when used for the prevention and cessation of SE.

## 1 INTRODUCTION

Status epilepticus (SE) is a condition resulting either from the failure of the mechanisms responsible for seizure termination or from the initiation of mechanisms, which lead to abnormally prolonged seizures.^1^ The annual incidence of SE in Europe is 9–36 per 100 000 adults,^2^ and globally, 1–74 per 100 000 adults^3^ and 18–23 per 100 000 children.^4^, ^5^

Approximately 40% of patients with SE have a history of epilepsy.^2^ The incidence of SE is relatively high in individuals with developmental and epileptic encephalopathies (DEEs) such as Dravet syndrome and Lennox–Gastaut syndrome.^1^, ^6^, ^7^, ^8^, ^9^, ^10^ Convulsive SE occurs in 47% of patients overall with genetic DEEs, and in 89% of patients with Dravet syndrome.^8^ Nearly 50% of children with Dravet syndrome aged <2 years have frequent SE episodes.^9^, ^11^ While the burden of SE is highest in early childhood, the risk of SE persists into adulthood.^12^

SE can be a serious and life‐threatening condition,^13^ and mortality may be as high as 30%–39%.^2^, ^14^ In children, SE can lead to significant motor disability and severe cognitive disability.^15^, ^16^ In a study of SE in infants and children (*n* = 339), 11% of patients died, 37% developed permanent neurologic sequelae, and 48% developed intellectual disability.^13^ Among patients with Dravet syndrome, SE is the second most common cause of death,^17^ with 25%–42% of deaths attributed to SE^18^; the risk of death due to SE is particularly high in early childhood.^12^ Moreover, the management of SE poses a considerable economic burden on patients and healthcare systems,^19^, ^20^ mainly due to the requirement for multiple antiseizure medications (ASMs), and frequent hospitalizations and intensive care unit admissions.^21^

To reduce mortality and morbidity associated with SE, therapeutic intervention aims to prevent SE (SE prevention), and once diagnosed, to rapidly terminate epileptic activity (SE cessation). For SE cessation, benzodiazepines are used as standard first‐line treatment, but treatment resistance is common (36% of patients with convulsive SE), requiring the use of additional ASMs.^22^, ^23^ If the seizures continue despite first‐line benzodiazepines and second‐line intravenous ASMs (e.g., levetiracetam or valproic acid), the SE is considered refractory to treatment and requires management in the intensive care unit.^24^ Super‐refractory SE occurs when the SE persists for ≥24 h after treatment with third‐line agents (e.g., anesthetic drugs, midazolam, thiopental, and ketamine) or recurs after withdrawal or reduction in third‐line agents.^24^ Super‐refractory SE is associated with high morbidity and mortality and considerable healthcare resource use and costs,^24^ and as such represents an unmet clinical need.

**FIGURE 1:**
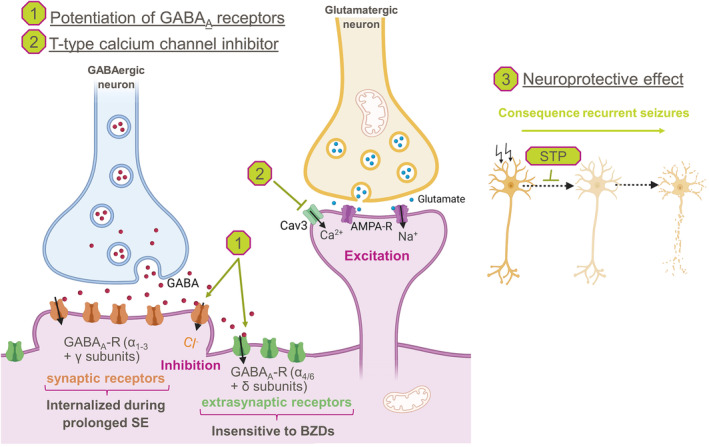
Mechanisms of action and biological properties of stiripentol (STP). (1) STP is a positive allosteric modulation of gamma‐aminobutyric acid (GABA)_A_ receptors,^28^, ^29^ targeting both γ‐containing (synaptic receptors) and δ‐containing (extra‐synaptic receptors) receptor subunits; (2) STP inhibits T‐type calcium channels: Cav3.1, 3.2 and 3.3^65^; and (3) STP has a neuroprotective effect by reducing the neuronal injury due to prolonged status epilepticus (SE).^32^ AMPA, α‐amino‐3‐hydroxy‐5‐methyl‐4‐isoxazole propionic acid; BZDs, benzodiazepines; R, receptor. Created with BioRender.com.

Stiripentol is a third‐generation ASM with a unique molecular structure.^25^, ^26^, ^27^ Stiripentol is a positive allosteric modulator of gamma‐aminobutyric acid (GABA)‐A receptors^28^ but does not interact with benzodiazepine binding sites at these receptors because of different subunit dependence (Figure 1).^29^ Given the pharmacodynamic profile of stiripentol relative to benzodiazepines,^28^, ^29^, ^30^ stiripentol is hypothesized to be effective in the treatment of SE, including benzodiazepine‐refractory SE, and preclinical data support this hypothesis.^31^ Additionally, stiripentol has a neuroprotective effect against neuronal injury caused by prolonged SE, which may help reduce the neurologic consequences of prolonged SE.^32^ Stiripentol is currently approved as an add‐on therapy in patients with Dravet syndrome: in combination with clobazam and valproate for refractory generalized tonic–clonic seizures in Europe^33^; and in combination with clobazam for the treatment of seizures in individuals aged ≥6 months of age who weigh ≥7 kg in the United States.^34^

Real‐world evidence suggests the effectiveness of stiripentol in the prevention of SE in patients with Dravet syndrome^35^ and to terminate SE.^4^ Thus, we aimed to review preclinical and clinical evidence describing stiripentol for the cessation of SE (including refractory or super‐refractory SE), and for the prevention of SE in patients with epilepsy syndromes predominantly associated with SE, such as Dravet syndrome and other DEEs.

## 2 MATERIALS AND METHODS

The text of this review follows the Preferred Reporting Items for Systematic Reviews and Meta‐Analysis (PRISMA) guidelines.^36^

### 2.1 Study selection criteria and search strategy

Studies were included if they reported data regarding the efficacy of stiripentol as treatment for the cessation of SE (including refractory or super‐refractory SE), or as treatment of epilepsy syndromes predominantly associated with SE; SE‐related outcomes had to be reported (e.g., cessation of the seizure, reduction in the frequency or duration of SE). The same inclusion/exclusion criteria were applied to human and animal studies alike. Reviews and commentaries/editorials were excluded from our data review, but if relevant were used to discuss our findings.

To identify studies for inclusion, we conducted an electronic literature search of the Cochrane Library (Wiley) and MEDLINE (access through PubMed [NLM]), using the search terms ‘stiripentol’ and ‘status epilepticus’ in all article fields and the BOOLEAN operator ‘AND’ (i.e., “(stiripentol) AND (status epilepticus)”) without restrictions on year or language. The last update search was conducted on September 14, 2023. Backward citation searching was also conducted manually by inspecting the reference list of published articles identified in the search.

Abstract books (gray literature) of relevant academic congresses/meetings were hand searched for the term “stiripentol” and then in the selected abstracts, for “status epilepticus.” Only meetings between July 2007 and September 2023 were included in the search, since stiripentol was first approved in Europe in 2007.^37^ The last search for full publications of any studies identified from meeting abstracts was conducted on October 25, 2023.

The electronic search results were entered into EndNote X7 (Thomson Reuters) reference management software for systematic and manual removal of duplicates if necessary. For the abstracts, the review for duplicates was done manually; those with a later publication in a scientific journal were discarded.

Initial screening of titles and abstracts was conducted to identify potentially relevant records according to the study inclusion/exclusion criteria, and the full text was then obtained to re‐apply these criteria. Literature searching and filtering were undertaken by two independent reviewers, and any disagreement was resolved via one of the authors, if required.

### 2.2 Data extraction

The following data were extracted from studies selected for inclusion in this review: population, disease, SE (definition, type, and duration before stiripentol treatment), stiripentol dosage regimen, prior medications, concomitant medications, efficacy outcomes (SE cessation when stiripentol was used to induce cessation of SE, and reduction in frequency/duration of SE episodes when used to treat epilepsy syndromes), hospitalizations (incidence), and safety (adverse events [AEs] and treatment modifications).

### 2.3 Outcomes and data analysis

The primary outcomes were as follows:

- the efficacy of stiripentol when administered as treatment for the cessation of SE;
- the efficacy of stiripentol when administered as treatment to reduce SE episodes in any epilepsy syndrome associated with predominant SE;
- the impact of SE treatment on hospitalization rate; and
- the AEs reported during treatment with stiripentol.

An additional exploratory outcome was treatment modifications.

The heterogeneity of study design and outcomes, the clinical characteristics of patient populations, and the reporting of SE outcomes precluded meta‐analysis (separately) of the human and animal data. Thus, studies and outcomes are described qualitatively below.

## 3 RESULTS

**FIGURE 2:**
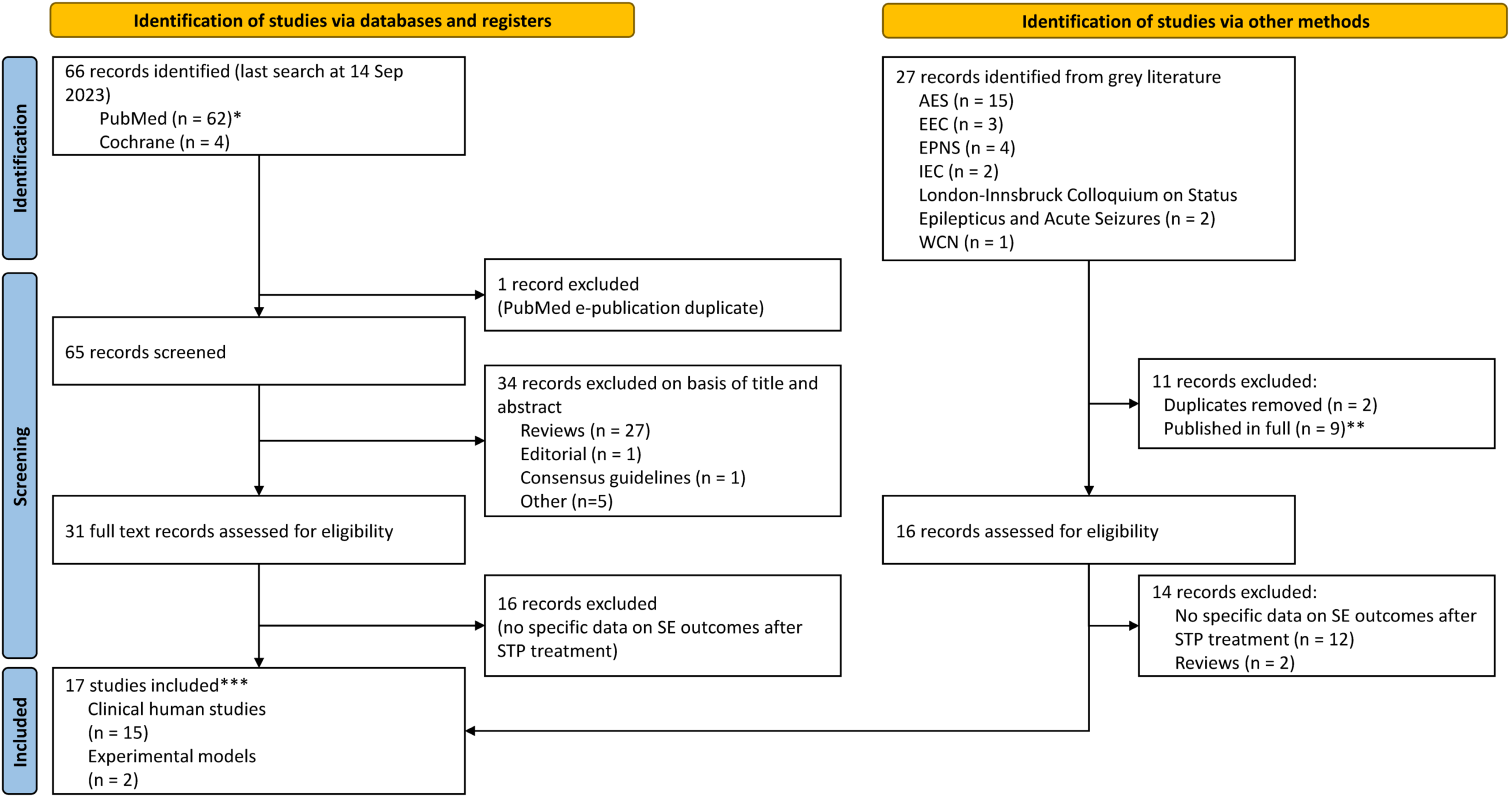
PRISMA flow chart. *Three articles found in PubMed were also found in the Cochrane search but are included here only in the PubMed search result; **One study was originally identified from the gray literature search but the full publication was later identified (Chiron et al. 2023^35^); ***Of which 16 were full publications and one was available only as a congress abstract (Chen et al. 2018 presented at AES 2018^40^). One of the full publications (Gil‐Nagel et al. 2023^43^) was originally identified in the hand search of congress abstracts. AES, American Epilepsy Society; EEC, European Epilepsy Congress; EPNSC, European Pediatric Neurology Society Congress; IEC, International Epilepsy Congress; SE, status epilepticus; STP, stiripentol; WCN, World Congress of Neurology.

Of 66 records identified from electronic database searches, 35 records were excluded after screening of the titles and abstracts and 31 records were selected for in‐depth review (Figure 2). Sixteen records were excluded after full‐text review because there were no data on SE outcomes after stiripentol treatment. Twenty‐seven abstracts of potential interest were identified from the proceedings of academic conferences, of which two were duplicates and nine had been subsequently published in full (Figure 2). Of the 16 remaining congress abstracts, only two met the study inclusion/exclusion criteria, of which one was found to be fully published during an ad hoc search in October 2023.

Seventeen studies were included in this review: two were animal studies,^31^, ^32^ and 15 were studies in humans.^11^, ^35^, ^38^, ^39^, ^40^, ^41^, ^42^, ^43^, ^44^, ^45^, ^46^, ^47^, ^48^, ^49^, ^50^ Results for one human study were available only as a congress abstract.^40^

### 3.1 Animal study characteristics and outcomes

Both studies used rodent models of SE, where SE was pharmacologically‐induced by lithium‐pilocarpine (Table 1).^31^, ^32^

**TABLE 1:** Experimental evidence (pre‐clinical data) published regarding the use of stiripentol for status epilepticus (SE) cessation.

Reference	Experimental model	Population	Stiripentol treatment	Outcomes
Auvin et al. (2013)^32^	Lithium‐pilocarpine model of SE	Wistar rats aged 75 days (‘mature brain’) or 21 days (‘immature brain’)	150, 250, or 350 mg/kg, 1 h before experiments	Reduced the incidence of SE at 350 mg/kg only in the immature brain Delayed the occurrence of SE at both ages (at a dose of 250 mg/kg in the immature brain and 350 mg/kg in the mature brain) Reduced the level of SE‐induced cell injury in the hippocampus
Grosenbaugh & Mott (2013)^31^	Lithium‐pilocarpine model of SE	Young and adult (15–23 or 57–63 day‐old) male Sprague–Dawley rats	10–1000 mg/kg at the onset of stage 3 behavioral convulsions (brief SE)	SE cessation with median effective dose of 100 mg/kg in young animals vs 377 mg/kg in adult animals Effectiveness also observed in SE resistant to DZP (i.e., prolonged SE) with similar effective doses

Abbreviations: DZP, diazepam; h, hour.

Stiripentol stopped SE when administered at the onset of behavioral seizures (e.g., twitching, head nodding, repetitive chewing, immobility and forelimb clonus without rearing)^31^ and reduced the incidence and latency of SE in rodents treated 1 h before induction of SE.^32^ The latter paper also revealed that stiripentol had neuroprotective effects against neuronal injury due to prolonged SE. Stiripentol was more effective in younger than older animals in both studies.^31^, ^32^ Stiripentol remained effective during prolonged SE (more than 45 min), when seizures had become resistant to benzodiazepine treatment.^31^

### 3.2 Human studies

Fifteen studies in humans were included for analysis (Figure 2). A total of 474 individuals were included in the human studies (aged 1.1–78 years). Twelve studies investigated stiripentol treatment for DEEs (Dravet syndrome in 11/12 studies): One was a case report,^38^ two were case series,^38^, ^40^, ^50^ one was a cross‐sectional study,^42^ six were retrospective studies,^35^, ^39^, ^41^, ^43^, ^46^, ^49^ and two were prospective studies^11^, ^44^ (Table 2). Three studies investigated stiripentol for the cessation of super‐refractory SE, including a case report and two case series.^45^, ^47^, ^48^

**TABLE 2:** Study design, patients and treatment history of human studies of stiripentol (STP) for preventing status epilepticus (SE)^a^ in patients with epilepsy syndromes and for the cessation of SE.

Reference	Study design (country)	No. of pts	Diagnosis	Age^b^	Treatment history
STP for prevention of SE					
Alhakeem et al. (2018)^38^	Retrospective case series (Saudi Arabia)	3	*SLC13A5*‐related epileptic encephalopathy and history of SE leading to ED visits/hospital admissions >6 times/y	NR	Numerous prior ASM, including PB, CLN, TPM, CBZ, and LEV
Balestrini and Sisodiya (2017)^39^	Retrospective clinical audit (United Kingdom)	13	DS (8 with episodes of SE)	Mean (SD) age when STP started: 26 (12) y [range 12–47 y]	Mean no. prior ASM: 9 [range 3–15]
Chen et al. (2018)^40^ *abstract*	Case series (China)	5	*SCN1A*‐related DS	Age range: 1.3–6.8 y	Mean no. prior ASM: 6 [range 2–10]
Chiron et al. (2018)^41^	Retrospective (France)	40	DS	Median [range] age when STP started:^c^ *V1*: 5.1 [0.8–17.7] y (*n* = 33) *V2 & V3*: 6.3 [0.8–20.9] y (*n* = 40)	All pts were treatment‐experienced prior to starting STP
Chiron et al. (2023)^35^	Retrospective (France)	131	DS	Median [range] age:^d^ *Baseline*: 1.1 [0.3–2.0] y *V1*: 1.4 [0.8–2.5] y *V2*: 3.4 [1.2–7.0] y	NR
De Liso et al. (2016)^42^	Cross‐sectional cohort (France)	54^e^	DS	Mean age at start of STP: 1.7 y [range 0.25–6 y] (*n* = 49)^g^	STP (*n* = 2)
Gil‐Nagel et al. (2023)^43^	Retrospective (Spain)	82^f^	DS (*n* = 55) Other DEEs (*n* = 26)	Median [IQR] age at start of STP: 5.2 [2.9, 12.7] y	Median no. of ASM: 3 [range 1–6] (*n* = 82)
Inoue et al. (2009)^44^	Prospective, observational, open‐label (Japan)	23^g^	DS	Median [range] age: *Group 1 (n = 15)*: 3 [1–8] y *Group 2 (n = 8)*: 19 [13–22] y	At least 1 prior ASM
Myers et al. (2018)^11^	Prospective, observational open‐label 12‐year cohort (Australia)	41	DS (27 pts with history of SE)	Median [range] age at start of STP: 5.6 [0.7–22 y]	NR
Thanh et al. (2002)^46^	Retrospective cohort (France)	46	DS	Median age: 2 y	VPA and CLB^h^
Wirrell et al. (2013)^49^	Retrospective cohort (USA)	82	DS History of SE (55% of pts)	Median [Q1, Q3] age at start of STP: 6.9 [4.2, 10.4] y	Median [Q1, Q3] no. of ASM: 7.0 [6.0, 9.0]
Yildiz et al. (2019)^50^	Case series (Turkey)	21^i^	DS History of CSE: 11/18 pts treated with STP	Mean [range] age: 8.2 [5.4–15] y	NR
STP for SE cessation					
Strzelczyk et al. (2015)^45^	Retrospective case series (Germany)	5	SRSE^j^ CSE (*n* = 2), NCSE (*n* = 3) History of seizures/SE (*n* = 4)	Median [IQR] age: 78 [11] y	Median [IQR] no. of prior ASM: 6 [1]
Uchida et al. (2017)^47^	Case report (Japan)	1	SRSE^j^	Age: 20 y	*Prior ASM*: VPA, CBZ, PHT, CLN, PB, LEV *Anesthetics*: MDZ, PRO, THY *KD*
Uchida et al. (2018)^48^	Case series (Japan)	5	SRSE with cross sensitivity^k^ No history of seizures (*n* = 5)	Mean [range] age: 37.8 [18–65] y	Mean [range] of ASMs: 9 [6–11]^h^ Cross sensitivity to ASM demonstrated prior to STP initiation

Abbreviations: ASM, antiseizure medication(s); CBZ, carbamazepine; CLN; clonazepam; CSE, convulsive status epilepticus (SE); DEES, developmental and epileptic encephalopathies; DS, Dravet syndrome; ED, emergency department; h, hours; IQR, interquartile range; KD, ketogenic diet; LEV, levetiracetam; MDZ, midazolam; min, minutes; mo, month(s); NCSE, non‐convulsive SE; no., number; NR, not reported; PB, phenobarbital; PHT, phenytoin; PRO, propofol; pts, patients; Q, quartile; RSE, refractory SE; SD, standard deviation; SRSE, super‐refractory SE; STP, stiripentol; THY, thiamylal; TPM, topiramate; VPA, valproate; y, year(s).

^a^ The definition of SE was specified in eight studies; in five of these, SE was defined as a seizure lasting ≥30 min,^35^, ^42^, ^43^, ^46^, ^49^ but Gil‐Nagel et al. included in the definition of SE ‘a series of epileptic seizures during which function is not regained between ictal events in a 30 min period’^43^; in the studies of SE cessation,^45^, ^47^, ^48^ SE was defined as continuous epileptic seizures or recurrent seizures without recovery of consciousness for more than 5 min.

^b^ Age is age at study entry, unless specified otherwise. Age is reported here in years (y), converting from months in the original publication if necessary.

^c^ Chiron et al. 2018 reported on treatment during childhood, and efficacy and safety outcomes for the following three time points: last visit before 15 years' of age (*V1*; *n* = 33), last visit before adulthood (*V2*; *n* = 40), and the last visit in adulthood (*V3*; *n* = 40).^41^

^d^ Chiron et al. 2023 provided data from three follow up visits: at STP initiation (baseline), after <6 months' therapy (here designated *V1*), and at the last visit before 7 years' of age (i.e., after long‐term therapy, here designated *V2*).^35^

^e^ 49/54 pts (91%) received treatment with STP.^42^

^f^ SE efficacy data were available only for the subgroup of pts who had ≥1 SE episode in the month prior to STP initiation (*n* = 12).^43^

^g^ Twenty‐five pts were enrolled but data for only 23 pts were reported (2 excluded because of STP discontinuation/dose reduction). SE data were reported as SE or cluster seizures. Group 1 were younger patients, and Group 2 were older patients.^44^

^h^ Patients continued ASMs when STP was initiated.^46^, ^48^ It is not clear from the source publication of Uchida et al. 2018 whether this count of ASMs included or excluded STP.^48^

^i^ Eighteen of the 21 pts in this case series were treated with STP.^50^

^j^ SRSE was defined in the German and Japanese studies as SE that continued for ≥24 h after initiation of general anesthetic treatment, or recurred after reduction or withdrawal of anesthesia^45^, ^48^; SRSE was not formally defined in the case report from Japan but epileptic activity was not suppressed by prior treatments (ASMs, general anesthetics, KD).^47^ Cross sensitivity was defined as the recurrence or sequential reactivation of rash from different ASMs.^48^

^k^ Only data for the 5 patients with cross sensitivity are included in this table; all were diagnosed with encephalitis.^48^ Clinical characteristics of patients with SRSE without cross sensitivity were also reported for 5 other patients, but these patients are not included in this table because data on treatments or outcomes were not reported by the study authors.^48^

**FIGURE 3:**
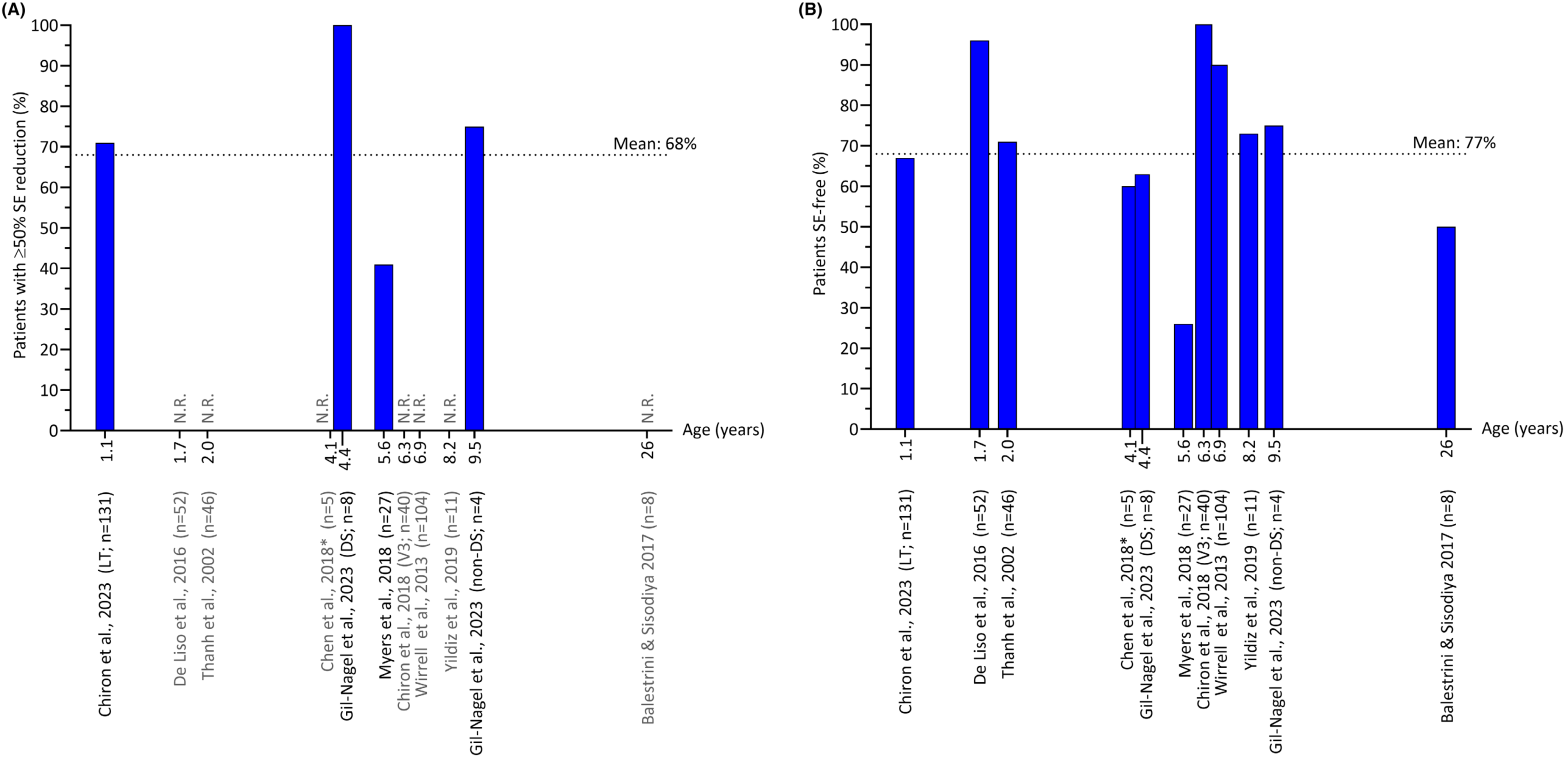
The effectiveness of stiripentol (STP) for the treatment of Dravet syndrome (DS) or non‐DS developmental and epileptic encephalopathies by patient age shown as (A) the proportion of patients with a ≥50% reduction in frequency of status epilepticus (SE) and (B) the proportion of patients who became SE‐free (100% reduction).^11^, ^35^, ^39^, ^40^, ^41^, ^42^, ^43^, ^46^, ^49^, ^50^ Age was plotted using log_10_ median values, except for the following studies (where log_10_ mean values were used): Balestrini & Sisodiya 2017,^39^ Chen et al. 2018 (mean age was derived from the range reported in the original publication),^40^ De Liso et al. 2016,^42^ and Yildiz et al. 2019^50^ (also see Table 2). Age was recorded at the time of stiripentol treatment initiation in six studies^11^, ^39^, ^41^, ^42^, ^43^, ^49^ and at baseline/study entry (unless specified otherwise) in five studies.^35^, ^40^, ^46^, ^50^ Age data from Gil‐Nagel et al. 2023^43^ are reported in this figure separately for patients with DS versus those with non‐DS developmental and epileptic encephalopathies (versus in Table 3, where age data are presented for all patients in this study). The first author, year of publication, and number of patients are shown below the x‐axis. Data are only for those patients who had SE prior to STP initiation (i.e., not necessarily the total number of patients in the study). The mean proportions of (A) 68% and (B) 77% were hand calculated (total number of patients (A) with ≥50% SE reduction or (B) SE–free divided by the total number of patients presenting with SE before STP treatment). Data from Chiron et al. 2018^41^ are reported for visit 3 (*V3*) and from Chiron et al. 2023^35^ for the long‐term (LT) follow‐up time point. Data from Inoue et al. 2009,^44^ where SE and seizure clustering results were reported together, and from Alhakeem et al. 2018,^38^ where patient age was not reported, are excluded from this figure. In (B), SE‐free data from Wirrell et al. 2013^49^ included in this figure were hand calculated based on data reported by treatment subgroup in the original publication (see Table 3). *Data are available only as an abstract.^40^ N.R., not reported.

Out of the 12 studies, 10 included patients with Dravet syndrome,^11^, ^35^, ^39^, ^40^, ^41^, ^42^, ^44^, ^46^, ^49^, ^50^ one included patients with Dravet syndrome and other DEEs,^43^ and one study (a case series) included patients with *SLC13A5*‐related DEEs^38^ (Table 2). Overall, most patients in these 12 studies were pediatric patients at the time stiripentol treatment was initiated (Table 2; Figure 3). Most study participants had previously received numerous ASMs (range 1–15) (Table 2).

In the three studies of patients (*n* = 11) treated with stiripentol to stop SE (acute treatment), all patients were adults who had previously received multiple ASMs (Table 2).^45^, ^47^, ^48^ In the case report from Japan, seizure history was not reported, although the patient was diagnosed with anti‐*N*‐methyl‐D‐aspartate receptor (NMDAR) encephalitis.^47^ In the two case series of patients requiring SE cessation, no patients had Dravet syndrome, four of 10 (40%) patients had a history of seizures,^45^, ^48^ and the clinical diagnoses were encephalitis,^47^, ^48^ congenital hemimegalencephaly, remote hemorrhage or remote ischemia.^45^

### 3.3 Human study outcomes

#### 3.3.1 Prevention of SE in epilepsy syndromes with stiripentol

A reduction in SE events after stiripentol initiation was reported in ≥26% of patients with Dravet syndrome or DEEs (Table 3),^11^, ^35^, ^38^, ^39^, ^40^, ^43^, ^44^, ^46^, ^49^ and the percentage of patients with a ≥50% reduction in SE from baseline varied between 41% and 100% (Figure 3A).^11^, ^35^, ^43^ Additionally, between 26% and 100% of patients became SE‐free after starting stiripentol treatment (Figure 3B, excluding data from Inoue et al. 2009 where SE and seizure clustering results were reported together, and Alhakeem et al. 2018 where patient age was not reported).

**TABLE 3:** Clinical evidence for the effectiveness of stiripentol (STP) in preventing status epilepticus (SE) when administered as a chronic medication in patients with epilepsy syndromes and when used as treatment for the cessation of SE in patients with super refractory SE (SRSE).^a^

Reference	No. of pts	STP treatment		Co‐administered ASM(s)	Effectiveness			
		Dosage	Duration		≥50% SE reduction^b^	Duration of SE	SE‐free	Other measure(s)
STP for SE prevention								
Alhakeem et al. (2018)^38^	3	Dose escalation over 1 mo to 40 mg/kg/d	18 mo	TPM CBZ			3/3 pts (100%)	
Balestrini and Sisodiya (2017)^39^	13	250–3000 mg/d Max. dose (mean): 1604 mg/d	Mean [range] 42 [3–139] mo	1–4 ASM			4/8 pts (50%) (mean [range] follow‐up: 43 [3–139] mo)	
Chen et al. (2018)^40^ *abstract*	5	20–40 mg/kg/d	4 mo	VPA and/or CLB (*n* = 3)		Decreased in 2/5 pts (40%)	3/5 pts (60%)	
Chiron et al. (2018)^41^	40	Median dose *V1*: 39 mg/kg/d *V2*: 34 mg/kg/d *V3*: 25 mg/kg/d	Median *V1*: 9 y *V2*: 11 y *V3*: 18 y	*V1*: CLB (*n* = 33) VPA (*n* = 32) TPM (*n* = 16) *V2, V3*: CLB (*n* = 40) VPA (*n* = 39) TPM (*n* = 20)			*V1*: 28/31 pts (90%)^c^ *V2*: 33/37 pts (89%)^c^ *V3*: 37/37 pts (100%)^c^	Pts with SE:^c^ B/line (aged <15 y): 50% *V1*: 3/31 *V2*: 4/37 *V3*: 0/37
Chiron et al. (2023)^35^	131	Median [Q1, Q3] dose^d^ *V1*: 50 [44.6, 63.2] mg/kg/d (*n* = 107) *V2*: 50 [40, 71.1] mg/kg/d (*n* = 100)	Median [Q1, Q3] *V1*: 3.7 [3, 4.9] mo *V2*: 27.8 [17.8, 39.1] mo	VPA (92–99%)^e^ CLB (95–97%)^e^ Other (13–24%)^d^	*V1*: 61% *V2*: 71%		*V1*: 55% *V2*: 67%	
De Liso et al. (2016)^42^	54	Mean [range] 42 [35–50] mg/kg/d^g^	~10 years^f^	At end of follow‐up (*n* = 43): VPA + CLB (*n* = 43) + ≥1 of: TPM (*n* = 20) LEV (*n* = 6) CLN (*n* = 5) ZNS (*n* = 4) Br (*n* = 1)		Seizures >5 min: 2/52 pts (3.8%)^g^	96% of pts^g^	
Gil‐Nagel et al. (2023)^43^	82^h^	Median [IQR] *DS (n = 55)*: 38.9 mg/kg [23.9, 48.1] *Other DEEs (n = 27)*: 30.0 mg/kg [19.2, 44.1]	Median [range] *DS (n = 55)*: 35.9 [0.8–164] mo *Other DEEs (n = 26)*: 17.0 [0.3–62.9] mo *p* < 0.001 DS vs other DEEs	*DS (n = 55)*: VPA 92.7% CLB 65.5% TPM 30.9% LEV 23.6% *Other DEEs* (*n* = 27): VPA 74.1% CLB 40.7% LEV 22.2% CLN 22.2%	11/12 pts (91.6%)^h^ No significant difference between DS (8/8 pts, 100%) vs other DEEs (3/4 pts, 75%)		8/12 pts (66.7%) No significant difference between DS (5/8 pts, 63%) vs other DEEs (3/4 pts, 75%)	
Inoue et al. (2009)^44^	23^i^	Mean [range] *Group 1*: 59.0 [30–100] mg/kg/d *Group 2*: 1469 [500–3000] mg/d	NR	VPA (*n* = 22) CLB (*n* = 11) Br (*n* = 5) PB (*n* = 5) Combinations with >3 drugs: *n* = 9				Reduction in frequency of SE or seizure clustering: 6/23 pts (26%) Freedom from SE or seizure clustering: 1/23 pts (4.3%)
Myers et al. (2018)^11^	41	Dose: Gradually titrated up to 67 mg/kg/d (max. 4 g/d)	Median [range] 37 [2–141] mo	CLB (80%) VPA (71%) TPM (61%) Mean [range] no. of co‐administered ASM: 2.8 [2–5]	11/27 pts (41%)		7/27 pts (26%)	
Thanh et al. (2002)^46^	46	50–100 mg/kg/d	Median [range] 34.8 [2–66] mo	VPA and CLB		*B/line*: Group I: 10 pts, 30 min to 10 h Group II: 18 pts, 30 min to 5 h *After treatment*: Group I: 4/10 pts, transitory 30–45 min, then free Group II: 5/18 pts, 30–75 min	30/42 pts (71%)	Reduction in number of SE episodes: 54% Better response in the youngest pts (<2 y)
Wirrell et al. (2013)^49^	82	Median [Q1, Q3] dose: 30 [22, 43] mg/kg/d	Median [Q1, Q3] 22.3 [9.9, 43.2] mo	VPA CLB		Duration of longest SE episode (mean):^j^ *B/line*: 186 min *After treatment*: STP + CLB 60 min STP + VPA 105 min STP + CLB + VPA 201 min	*B/line*: 38/82 pts (46%) *After treatment*: STP^j^ 6/6 pts (100%) STP + CLB 34/35 pts (97%) STP + VPA 12/15 pts (80%) STP + CLB + VPA 43/48 pts (90%)	
Yildiz et al. (2019)^50^	21^k^	Mean [range] MTD: 33.2 [31–40] mg/kg/d (*n* = 18)	Mean [range] 41.2 [24–64] mo	No. of ASM in total: *Two* 3/21 pts (14%) *Three* 13/21 pts (62%) *Four* 5/21 pts (24%)			8/11 pts (73%) at mean of 44 mo after STP initiation	
STP for SE cessation^l^								
Strzelczyk et al. (2015)^45^	5	*Initial*: 2–5 g/d *Final*: 4–6 g/d Dose titration period: 2–3 d Median [IQR] latency from SE onset to STP initiation: 39 [16] days	NR	LOR (*n* = 5)^m^ VPA (*n* = 2) LEV (*n* = 4) TPM (*n* = 3) LCS (*n* = 3) LTG (*n* = 1) PER (*n* = 2)	NA	NA	SE cessation within 2.5–4 d (*n* = 3)	NA
Uchida et al. (2017)^47^	1	Loading dose: 500 mg/d Maintenance dose: 2500 mg/d (reached after 2 mo)	NR	KD	NA	NA	SE cessation by 2 mo after starting STP	NA
Uchida et al. (2018)^48^	5^n^	Loading dose: 500–1000 mg/d, titrated by 500 mg/wk	NR	Range: 1–2 per pt CLB (*n* = 3) LCS + ZNS (*n* = 1) TPM + PER (*n* = 1)	NA	NA	Mean [range] time to SE cessation: 30.8 [18–46] d	NA

*Note*: See Table 2 for study design details.

Abbreviations: ASM, antiseizure medication(s); b/line, baseline; Br, bromide; CBZ, carbamazepine; CLB, clobazam; CLN, clonazepam; CSE, convulsive SE; d, days; DEEs, developmental and epileptic encephalopathies; DS, Dravet syndrome; ED, emergency department; h, hours; IQR, interquartile range; LCS, lacosamide; LEV, levetiracetam; LOR, lorazepam; LTG, lamotrigine; max., maximum; min, minutes; mo, month(s); MTD, maximum tolerated dose; NA, not applicable; no., number; NR, not reported; PB, phenobarbital; PER, perampanel; pts, patients; Q, quartile; TPM, topiramate; VPA, valproate; wk(s), week(s); y, year(s); ZNS, zonisamide.

^a^ See Table 2 for the definitions of SE and SRSE.

^b^ This refers to a ≥50% decrease in SE frequency vs baseline.

^c^ Chiron et al. 2018 reported on treatment during childhood, and efficacy and safety outcomes for the following three time points: last visit before 15 years' of age (*V1*; *n* = 33), last visit before adulthood (*V2*; *n* = 40), and the last visit in adulthood (*V3*; *n* = 40).^41^ SE outcomes were reported as the proportion of pts with SE (‘Other measures’); these data were converted to percentage pts ‘SE‐free’ for inclusion also in the ‘SE‐free’ column.

^d^ Chiron et al. 2023 provided data from three follow up visits: at STP initiation (baseline), after <6 months' therapy (here designated *V1*), and at the last visit before 7 years' of age (i.e., after long‐term therapy, here designated *V2*).^35^

^e^ Range of percentage of patients across baseline, *V1* and *V2*.^35^

^f^ 49/54 pts (91%) initiated treatment with STP and 47/49 STP recipients remained on treatment at the end of follow‐up (mean age at end of follow‐up in all pts was 10 y 1 mo; *n* = 54).^42^

^g^ The authors defined SE as CSE lasting >30 min, and reported that 96% of pts had clonic or tonic–clonic seizures of <5 min duration and the last SE episode occurring before the age of 4 years.^42^

^h^ SE efficacy for the subgroup of pts who had ≥1 SE episode in the month prior to STP initiation are reported (*n* = 12); in the original study publication, the duration of STP treatment in pts with other DEEs was reported as 17.3 mo in the text vs 17.0 mo in a table.^43^

^i^ Twenty‐five pts were enrolled but data for only 23 pts were reported (2 pts were excluded because of STP discontinuation/dose reduction). SE was not reported separately from seizure clustering.^44^ One of the two excluded patients was a 5‐year‐old girl in whom STP was re‐introduced after she was withdrawn from the study, and SE disappeared completely.

^j^ SE results are reported by main concomitant treatment (CBL, VPA) but does not imply that pts did not receive other ASMs. Six children were treated with STP without CLB or VPA but did receive a median of 2 other ASMs.^49^ The SE‐free rate was calculated from data on the incidence of SE in the source publication (b/line: 44/82 pts (54%) vs after treatment: STP, 0/6 pts (0%); STP + CLB, 1/35 pts (3%); STP + VPA, 3/15 pts (20%); STP + CLB + VPA, 5/48 pts (10%)). Note that the treatment group denominators sum to 104 vs a total study population size of 82 patients.

^k^ Eighteen of the 21 pts in this case series were treated with STP.^50^

^l^ STP was administered for acute SE cessation via a nasogastric tube 2–3 times daily, using STP powder for oral suspension in the study by Strzelczyk et al.^45^; route of administration was not reported in the two Uchida et al. publications.^47^, ^48^

^m^ All patients received LOR in place of CBZ to better assess the effect of STP on refractory SE (i.e., without an interaction with CLB).^45^ One patient was placed on a ketogenic diet in addition to receiving ASMs. All patients received anesthetic drugs (1–4 different anesthetics) for >24 h, including either propofol, thiopental, midazaolam, or ketamine, or combinations thereof.

^n^ Only data for the 5 patients with cross sensitivity are included here.^48^ All patients received anesthetic drugs (2–3 different anesthetics) for >24 h, including midazaolam, propofol or thiopental, or combinations thereof.

A retrospective study including 82 patients with Dravet syndrome (Wirrell et al.^49^) reported results by concomitant ASM: The proportion of patients with SE decreased from 54% in the year prior to stiripentol treatment initiation to 3%, 20%, and 10% after receiving stiripentol plus clobazam, stiripentol plus valproate, or stiripentol plus clobazam and valproate, respectively. All patients who received stiripentol as add‐on therapy to ASMs (excluding those who received the drug in combination with clobazam and valproate; a small group of only six patients) had no further SE after starting stiripentol.^49^

The duration of follow‐up for assessing the effect of stiripentol treatment (i.e., stiripentol treatment duration) varied widely across studies, from 4 to 42 months to up to 18 years (Table 3). The effectiveness of stiripentol in reducing SE was seen over the long term, with a possible association with age at treatment initiation. In a cross‐sectional study, the majority of patients received the triple combination of stiripentol plus clobazam and valproate for ~10 years (43/53 patients [81%]), and all except 2 of the 52 patients in the overall population experienced seizure duration of <5 min.^42^ In the prospective observational study by Myers et al., 27 of the 41 patients included in the study had SE in the 3 months prior to starting stiripentol; 11 of these 27 patients (41%) experienced a reduction from the time of stiripentol initiation of at least 50% in SE event frequency over a median treatment duration of 3 years (range 2 months to 12 years); a reduction of ≥90% was seen in 9/27 (33%) patients.^11^ While the median age at which stiripentol was initiated in these 27 patients was not reported, the youngest was aged 11 months and the eldest 22 years.^11^ Long‐term data revealed a sustained reduction in convulsive SE frequency in the study by Thanh et al., especially in those patients starting stiripentol before the age of 2 years.^46^ The greatest reduction was observed in the youngest patients.^46^ This age‐dependent effect was also observed in an open‐label multicenter study by Inoue et al., in which the frequency of SE decreased in 5/15 patients who started stiripentol between the age of 1 and 8 years (median 3 years) versus 1/8 patients who started between the age of 13 and 22 years (median 19 years).^44^ In one of the largest studies to date (*n* = 131), that collected data across a 30‐year time period from patients treated with stiripentol, and followed up, for different durations, but in whom stiripentol treatment was initiated prior to the age of 2 years.^35^ Sixty‐one percent of patients experienced a ≥ 50% reduction in SE events over the short term (median of 4 months after treatment initiation), and this benefit improved as treatment continued, with 71% being responders a median of 2 years after treatment initiation (Table 3).^35^ Notably, this study defined SE as seizures lasting >30 min.

Two studies reported a reduction in the duration of SE with stiripentol (Table 3).^40^, ^49^ One of these studies reported only that the duration of SE was reduced in 2/5 patients.^40^ In the other (abovementioned Wirrell et al. study^49^), the mean duration of the longest SE event in the year prior to stiripentol initiation was 186 min, and this was reduced to 60–105 min after stiripentol initiation among patients also treated with clobazam or valproate.^49^ SE duration was 201 min after stiripentol initiation in the group receiving both clobazam and valproate.^49^

Most studies reported that at least some patients no longer experienced SE after starting stiripentol treatment (Table 3).^11^, ^35^, ^38^, ^39^, ^40^, ^41^, ^43^, ^44^, ^46^, ^49^, ^50^ In three studies, all patients became SE‐free (i.e., 100% of patients): 37/37 patients at their last follow‐up in adulthood in patients who had started stiripentol in childhood or adolescence,^41^ 6/6 children after 6 months' stiripentol treatment in the subgroup who received other ASMs (but not valproate or clobazam),^49^ and 3/3 patients in a case series.^38^

Stiripentol was administered in doses from 20 to 50 mg/kg/day in combination with at least one other ASM, usually valproate and/or clobazam (Table 3). Treatment with stiripentol was maintained for long periods of time—usually more than 2 years. The longest duration of treatment was that reported by Chiron and colleagues,^41^ a median of 18 years. Moreover, treatment with stiripentol allowed for a reduction in the number of ASMs used to control seizures. For example, in the Gil‐Nagel et al. study, 16.7% of patients discontinued at least one ASM, and 9.2% discontinued at least two ASMs.^43^

Few studies reported stiripentol effectiveness in relation to emergency department (ED) visits and/or hospital admission (Table S2).^35^, ^38^, ^39^, ^49^ Two small studies reported that after starting stiripentol, no patients attended ED or were admitted to hospital.^38^, ^39^ In the two larger studies,^35^, ^49^ the frequency of ED visits/hospital admissions decreased during stiripentol treatment. One of these studies also reported rescue medication use,^49^ noting a reduction in frequency of rescue medication use of between 50% and 100% (Table S2).

#### 3.3.2 Acute treatment of SE with stiripentol

The duration of super‐refractory SE before stiripentol was initiated ranged from 20 to 82 days in the two case series,^45^, ^48^ and was not reported in the case report.^47^ Not all patients had a prior history of seizures (where reported), and various other clinical diagnoses had been made (e.g., (encephalitis)) in patients experiencing this super‐refractory SE (see “Human studies” section, above). Stiripentol was administered as add‐on therapy to other ASMs, including lorazepam, valproate, levetiracetam, topiramate, lacosamide, clobazam, perampanel, or lamotrigine (Table 3), and general anesthetics. Two patients were also treated with a ketogenic diet: the patient case report from Uchida and colleagues,^47^ and one of the five patients in the case series from Strzelczyk and colleagues.^45^ Notably, stiripentol was used without concomitant clobazam in patients with convulsive or non‐convulsive SE.^45^, ^47^ The patient treated by Uchida and colleagues had received treatment with general anesthetics (midazolam, propofol and thiamylal) prior to initiating treatment with a ketogenic diet and stiripentol.^47^ Anesthetics were administered, usually for a duration of >24 h, as concomitant therapy to ASMs in all five patients in the case series reported by Strzelczyk and colleagues^45^ and the five patients in the case series reported by Uchida and colleagues,^48^ including propofol, thiopental, midazolam or ketamine or combinations thereof.

SE cessation, defined as attenuation of status in electroencephalography followed by resolution, was observed in the single patient case report and 5/5 patients in the case series from Japan,^47^, ^48^ and in 3/5 patients in the case series in Germany.^45^ In this series, a rapid SE cessation (2.5–4 days) was also reported.^45^ The time to SE cessation was 0.6–2 months in the case report and the other case series.^47^, ^48^

### 3.4 Safety of stiripentol in SE prevention and acute treatment

Overall, the most common stiripentol‐related AEs reported were loss of appetite, weight loss, anorexia, sleep disturbance, and irritability/excitability (Table 4). The percentage of patients with AEs ranged between 17% and 73% (Figure S1). In most cases, AEs resolved after dose modification of concomitant ASM(s) and/or stiripentol, but few patients discontinued stiripentol due to an AE (Table 4).

**TABLE 4:** Adverse events (AEs) and discontinuations due to AEs reported with stiripentol (STP) treatment for patients with epilepsy syndromes or for the cessation of status epilepticus (SE).

Reference	No. of pts	Common AEs/AE profile	Discontinuation of STP due to AE
Stiripentol for DS or other DEEs			
Alhakeem et al. (2018)^38^	3	NR	NR
Balestrini and Sisodiya (2017)^39^	13	Anorexia/weight loss (*n* = 4), unsteadiness (*n* = 3), and tiredness/somnolence (*n* = 2), nausea (*n* = 1), abdominal pain (*n* = 1), diarrhea (*n* = 1), myelodysplasia (thrombocytopenia and neutropenia) (*n* = 1), behavioral disturbance (*n* = 1), and increased tremor (*n* = 1)	3/13 pts (23%)
Chen et al. (2018)^40^ *abstract*	5	Nausea, vomiting, anorexia, drowsiness, weary, ataxia, and excitability (all resolved within 1 mo)^a^	NR
Chiron et al. (2018)^41^	40	^b^ *V2*: STP‐related anorexia and bodyweight loss (1/18 pts) and STP‐related asymptomatic neutropenia (1/18 pts), resolving with STP dose reduction *V3*: 16 STP‐related events (anorexia/weight loss in 15 cases and myoclonus in 1 case), which resolved after reducing STP and/or VPA dose	NR
Chiron et al. (2023)^35^	131	Loss of appetite/weight (21% of pts), somnolence/sleep disorders (11%), agitation/irritability (7%) and hypotonia (5%) Most AEs resolved after dose reduction	3/131 pts (9.7%)
De Liso et al. (2016)^42^	49^c^	Fatigue, somnolence, and moderate anorexia	No pts needed to stop triple therapy because of AEs, although doses reduced in some pts^a^
Gil‐Nagel et al. (2023)^43^	82	Drowsiness (21.9% of pts), reduced appetite (18.3%), irritability (13.4%), gait instability (12.2%)	6/82 pts (7.3%)
Inoue et al. (2009)^44^	23	*Early follow‐up*:^d^ Loss of appetite (*n* = 8), drowsiness (*n* = 10), hyperactivity/irritability (*n* = 6), ataxia (*n* = 5), insomnia (*n* = 2) AEs disappeared after dose modification in most cases *Late follow‐up*:^d^ loss of appetite (*n* = 3), ataxia (*n* = 1)	Late follow up: 1/23 (4.3%)
Myers et al. (2018)^11^	41	Anorexia/weight loss (49% of pts), drowsiness/sedation (34%), behavioral changes (22%), neutropenia (12%), abdominal pain (10%), insomnia (10%), ataxia/unsteadiness (7%), drooling (7%), tremor/myoclonus (7%) Other AES in <7% of pts: vomiting, absence seizure increase, gamma‐glutamyl transferase elevation, hypoalbuminemia/edema, nightmares, recurrent pancreatitis, streptococcus‐induced toxic shock syndrome	NR^e^
Thanh et al. (2002)^46^	46	Loss of appetite (56% of pts) and weight loss (36%) AEs more severe in pts over 12 years of age (STP dosage could not be increased to 50 mg/kg/d)	3/46 pts discontinued STP due to AEs; 7/46 pts temporarily discontinued STP due to AEs before restarting STP at a lower dose
Wirrell et al. (2013)^49^	82	Sedation/somnolence (18.3% of pts), ataxia/balance disorder (4.9%), insomnia (3.7%), seizures/convulsions (3.7%)	5% of pts discontinued STP for AEs
Yildiz et al. (2019)^50^	21	Sedation (6/8 pts), ataxia (5/8 pts)	STP was ‘disrupted’ in 2/21 pts (9.5%) because of severe somnolence and an increase in seizure number^f^
Stiripentol for cessation of SE			
Strzelczyk et al. (2015)^45^	5	No STP‐related serious AEs	NR
Uchida et al. (2017)^47^	1	NR	NR
Uchida et al. (2018)^48^	5	No additional AEs after STP initiation	NR

Abbreviations: DEEs, developmental and epileptic encephalopathies; DS, Dravet syndrome; mo, month; NR, not reported; pts, patients; VPA, valproate.

^a^ Numerical data not reported.^40^, ^42^

^b^ Chiron et al. 2018 reported on treatment during childhood, and efficacy and safety outcomes for the following three time points: last visit before 15 years' of age (*V1*; *n* = 33), last visit before adulthood (*V2*; *n* = 40), and the last visit in adulthood (*V3*; *n* = 40).^41^

^c^ 49/53 patients received STP in this study.^42^ Safety data are for STP recipients.

^d^ The ‘early follow up’ period was during the first 4 weeks of STP add‐on therapy, and the ‘late follow up’ period was during long‐term treatment.^44^

^e^ A total of 8/41 (19.5%) pts discontinued STP for AEs or lack of efficacy.^11^ Data exclusively for discontinuations due to AEs was not reported.

^f^ Eighteen of the 21 pts in this case series were treated with STP.^50^ As indicated in the ‘Safety’ section of the table for this study, the authors stated that STP treatment was ‘disrupted’. It is not clear whether this was a treatment interruption or discontinuation.

Only two of the three studies that assessed stiripentol for SE cessation reported treatment tolerability (Table 4). There were no serious treatment‐related AEs reported in the German case series,^45^ and no new AEs observed after the initiation of treatment in the Japanese case series.^48^

## 4 DISCUSSION

This systematic review collated evidence from experimental and clinical studies on the effectiveness of stiripentol for the prevention and cessation of SE. Two settings for the use of stiripentol were reviewed: first, the use of stiripentol administered as a chronic ASM to treat epilepsy syndromes characterized by SE episodes, and second, as treatment for the cessation of epileptic activity and resolution of SE. In animal models of SE, stiripentol was effective in halting SE, including when seizures were resistant to benzodiazepines, and reducing the incidence of SE or delaying SE onset when given before induction of SE. In human studies, we found that add‐on stiripentol treatment reduced the frequency of SE in patients with Dravet syndrome and in some patients with other DEEs, and that stiripentol treatment resulted in SE cessation when initiated in adult patients with super‐refractory SE. Overall safety outcomes were favorable, with few patients discontinuing treatment with stiripentol.

The 15 human clinical studies included were heterogeneous, with wide variations in methodological processes, including in study design, interventions, outcome measures and follow‐up duration, and in patient inclusion/exclusion criteria, clinical characteristics and history. As such, conclusions drawn should be interpreted with caution. Nevertheless, given the scarcity of data in this patient population, and the rigorous approach in developing this review, the results comprehensively present all evidence to date on the effectiveness of stiripentol on SE outcomes.

One of the animal studies included in the current review found that stiripentol remained effective during prolonged SE, an effect not observed with diazepam.^31^ This specific effect can be attributed to at least two pharmacological properties of stiripentol: (i) the potentiation by stiripentol of δ‐subunit–containing GABA‐A receptors, which are not internalized in the cell during prolonged SE,^28^, ^51^ and (ii) stiripentol inhibition of Cav 3.2 and 3.3 calcium channels, expression of which is upregulated during or after SE in animal models of SE.^52^, ^53^

The majority of studies included in this review that provided data on SE prevention with add‐on stiripentol treatment enrolled patients with Dravet syndrome, which is in line with the approved indication of stiripentol, that is, for treatment‐refractory seizures in patients with Dravet syndrome. When used as a treatment for Dravet syndrome (or other DEEs), we found that stiripentol (usually in combination with valproate, clobazam, or both) helped to prevent SE. There was a reduction in frequency of SE episodes and many patients became SE‐free (26%–100%), including over the long term. In addition, stiripentol reduced the frequency of ED visits or hospitalizations.^35^, ^49^

Some studies reported a better response to stiripentol in younger patients with Dravet syndrome,^44^, ^46^ consistent with results from animal studies.^31^, ^32^ If the incidence of SE could be reduced by stiripentol during the first years of life in patients with Dravet syndrome, there is a possibility that serious sequelae of SE, such as cognitive delay and developmental regression, might be reduced or even prevented,^8^, ^43^, ^50^, ^54^ although there may be neurologic effects experienced by a patient due to the underlying etiology of their epilepsy syndrome. Further, reducing the frequency of SE, or halting SE altogether, may help to avoid or reduce the SE‐associated morbidity and mortality observed in this pediatric population.^8^, ^9^, ^55^, ^56^ However, there remains a lack of prospective clinical trials in children with Dravet syndrome or other DEEs evaluating the efficacy of stiripentol in preventing SE‐associated sequelae.

Only three case series were identified that provided data on the use of stiripentol to terminate SE.^45^, ^47^, ^48^ Given that all three of these studies (case reports/series; total *n* = 11) reported that add‐on stiripentol resulted in cessation of super‐refractory SE in almost all patients,^45^, ^47^, ^48^ this suggests that stiripentol could be considered for use in patients with refractory SE in palliative or life‐sustaining therapy settings, as already proposed in a recent systematic review.^57^ Recent data highlight the long‐term deleterious neurocognitive effects of SE in patients in a life‐sustaining therapy setting,^58^ and in our view underscores the importance of finding effective therapeutic options for these patients. Given that the data for the use of stiripentol for super‐refractory SE are limited to only three publications, and that patients were heavily pre‐treated with multiple anticonvulsants and anesthetics before stiripentol administration, further research would be required before recommendations to use stiripentol in this setting are formally adopted. Future research would need to include investigation of different stiripentol dosage regimens (e.g., loading dose, dose increments and duration of dose titration), since the study that reported the shortest duration until SE cessation (within 2.5–4 days of stiripentol treatment initiation^45^) utilized much higher stiripentol loading doses (2000–5000 mg/day vs. 500–1000 mg/day) and final daily doses (4000–6000 mg/day vs. 2500 mg/day) than the studies reporting a much longer time (up to 2 months^47^, ^48^) to SE cessation.

Our review highlighted the acceptable safety profile of stiripentol in children and adults as continuous treatment for Dravet syndrome. As may be expected in this patient population,^59^ combination treatment of ≥2 ASMs was common in the studies included in this review; thus, some of the AEs reported (loss of appetite or anorexia, weight loss, sleep disturbance, and irritability/excitability) are known AEs when stiripentol is given in combination with other ASMs such as clobazam (e.g., somnolence) or valproate (e.g., gastrointestinal AEs).^59^ Where reported in the studies included in our review to assess SE prevention, AEs resolved following a dose reduction of stiripentol and infrequently led to treatment discontinuation (Table 4). While certain combinations of ASMs can lead to an increase in AE intensity through drug–drug interactions, there may be rationale in administering both to exploit a pharmacodynamic drug–drug interaction leading to an increase in serum drug levels that may potentially augment the antiseizure effect (e.g., stiripentol and clobazam).^59^, ^60^ Dose adjustments may allow for minimization of AEs while maintaining the therapeutic advantage of the combination, as has been suggested for when clobazam is given in patients receiving stiripentol.^59^ Although data on SE cessation are limited to three case studies, stiripentol appears to be well tolerated for the cessation of SE in the hospital setting, although in this setting it may be difficult to discriminate stiripentol‐related AEs from other treatment‐related AEs and clinical factors.

Taken together, these data suggest that the use of stiripentol for the prevention of SE should be investigated in future prospective studies in pediatric patients with non‐Dravet DEEs characterized by frequent episodes of SE, and in a wider range of patients for the cessation of super‐refractory SE (e.g., pediatric patients, since current data on super‐refractory SE included in this review were only for adults). As mentioned earlier, SE is associated with significant morbidity and mortality,^2^, ^12^, ^13^, ^18^ and may have a negative impact on a patient's quality of life (QoL):^61^, ^62^ by preventing SE, it is hypothesized that stiripentol treatment may reduce morbidity, improve patient QoL and reduce SE‐associated mortality, but prospective studies would be required to examine the relationship between SE reduction/prevention and these outcomes (especially QoL, for both patients and families, which is not well studied). Similarly, the direct and indirect cost benefits of reductions in ED visits and hospitalizations should be specifically determined in future prospective stiripentol studies. A previous study in patients with Dravet syndrome reported that hospitalization costs accounted for a higher proportion of direct costs than ASMs (69% vs 24% of per patient direct costs), and among patients with treatment‐refractory seizures receiving adjunctive therapy with stiripentol and clobazam, a reduction in seizure frequency was associated with a 39% decrease in non‐ASM direct costs, mainly due to fewer inpatient admissions.^63^

Limitations of this review include the lack of a systematic assessment of the methodological quality of the included studies and the extreme heterogeneity (both clinical and methodological) across studies included in the review due to variability in study populations, interventions, and outcome measures, making it difficult to draw firm conclusions. In addition, there were variations between studies in how SE was defined, making it difficult to compare results across studies. There are also numerous limitations related to the quality of the included studies: (i) Most studies were observational studies, which makes it difficult to establish a causal relationship between stiripentol treatment and SE outcomes because observational studies are prone to confounding, which can lead to inaccurate estimates of the treatment effect; there may be confounding by indication, and confounding through the use of comedications and cotreatments, and by the age at which stiripentol is administered (since SE frequency tends to decrease with increasing age in patients with Dravet syndrome, as part of the natural history/evolution of the disease^64^), as well as due to the number and type of prior ASMs; (ii) retrospective studies and case reports are subject to selection bias; (iii) the relatively small number of patients in included studies (a characteristic of rare diseases) leading to imprecision in individual study results; (iv) the presence of positive reporting bias, where studies with positive results are more likely to be published than those with negative results, leading to an overestimation of treatment effect; and (v) the lack of robust randomized studies limits the applicability of the results of this review to inform clinical practice. It is important to keep these limitations in mind when interpreting the results of the systematic review. The design of future studies of stiripentol should ideally address these limitations, for example by conducting multicenter studies to increase patient sample size or to use appropriate statistical methods to adjust for confounding, to help improve the quality of evidence for stiripentol. A strength of this systematic review is the comprehensive systematic literature search that was conducted to identify all relevant studies, and as such, this review presents a comprehensive qualitative evaluation of all evidence to date on the effectiveness of stiripentol on SE outcomes.

In conclusion, clinical evidence suggests that stiripentol is a well‐tolerated and effective therapy that markedly reduces the frequency of SE, and possibly the duration of SE, in Dravet syndrome, and it should be introduced as early as possible in the disease course in order to limit the morbidity and mortality associated with SE episodes. In addition, data from a limited number of patients suggest that stiripentol may be a useful treatment in patients with DEEs and for the cessation of super‐refractory SE.

## AUTHOR CONTRIBUTIONS

The authors proposed the concept of this review and were involved in data evaluation/review and manuscript review/revisions. All authors have contributed equally to this work.

## CONFLICT OF INTEREST STATEMENT

NS has served on scientific advisory boards for GW Pharma, BioMarin, Biocodex, Arvelle, Marinus, and Takeda; has received speaker and/or consultancy honoraria from Eisai, Biocodex, Biomarin, Livanova, and Sanofi; and has served as a study investigator for Zogenix, Marinus, Biomarin, UCB, and Roche. SA is Deputy Editor for *Epilepsia*; has served as a consultant or received honoraria for lectures from Angelini Pharma, Biocodex, Biomarin, Eisai, Encoded, Jazz Pharmaceutics, Grintherapeutics, Neuraxpharm, Nutricia, Orion, Proveca, Stoke, Takeda, UCB Pharma, and Xenon; and has been an investigator for clinical trials for Eisai, Marinus, UCB Pharma, Takeda, and Xenon. AS has received personal fees and grants from Angelini Pharma (Arvelle), Biocodex, Desitin Arzneimittel, Eisai, Jazz (GW) Pharmaceuticals, Marinus Pharma, Precisis, Takeda, UCB Pharma (Zogenix), and UNEEG medical. FB has no conflicts of interest to disclose. VV has received honoraria and/or research funds from Angelini Pharma, Bial, Biocodex, Eisai, Jazz Pharmaceuticals, Neuraxpharm, Novartis, Nutricia, Takeda, UCB Pharma, and Xenon. ET has received personal fees from EVER Pharma, Epilog, Marinus, Arvelle, Angelini, Argenx, Medtronic, Bial‐Portela & Cª, NewBridge, GL Pharma, GlaxoSmithKline, Boehringer Ingelheim, LivaNova, Eisai, UCB, Biogen, Biocodex, Sanofi, Jazz Pharmaceuticals, and Actavis; and his institution has received grants from Biogen, UCB Pharma, Eisai, Red Bull, Merck, Bayer, the European Union, FWF Österreichischer Fond zur Wissenschaftsforderung, Bundesministerium für Wissenschaft und Forschung, and Jubiläumsfond der Österreichischen Nationalbank. We confirm that we have read the Journal's position on issues involved in ethical publication and affirm that this report is consistent with those guidelines.

## Supporting information


Appendix S1

## Data Availability

Data sharing is not applicable to this article as no new data were created or analyzed in this study.
