# Supplementary material for: Efficacy and safety of stiripentol in the prevention and cessation of status epilepticus: A systematic review
Source: Epilepsia Open. 2024 Oct 3;9(6):2017–36. doi: 10.1002/epi4.13036 (PMC11633682; doi:10.1002/epi4.13036)
Supplement: Supplementary file 1 — Appendix S1 [file EPI4-9-2017-s001.docx]

# SUPPORTING INFORMATION

## Efficacy and safety of stiripentol in the prevention and cessation of status epilepticus: a systematic review

**Authors:**Nicola Specchio^1^

Stéphane Auvin^2,3,4^

Adam Strzelczyk^5^

Francesco Brigo^6^

Vicente Villanueva^7^

Eugen Trinka^8,9,10^

**Affiliations:**^1^Neurology, Epilepsy and Movement Disorders Unit, Bambino Gesù Children's Hospital, IRCCS, Member of ERN EpiCARE, Rome, Italy

^2^Université Paris Cité, INSERM NeuroDiderot, Paris, France

^3^APHP, Robert Debré University Hospital, Pediatric Neurology Department, CRMR epilepsies rares, Member of ERN EpiCARE, Paris, France

^4^Institut Universitaire de France (IUF), Paris, France

^5^Goethe-University Frankfurt, Epilepsy Center Frankfurt Rhine-Main, Department of Neurology, University Hospital Frankfurt, Frankfurt am Main, Germany

^6^Department of Neurology, Hospital of Merano (SABES-ASDAA), Merano, Italy

^7^Refractory Epilepsy Unit, Neurology Service, Hospital Universitario y Politécnico La Fe, Member of ERN EpiCARE, Valencia, Spain

^8^Department of Neurology, Christian-Doppler University Hospital, Paracelsus Medical University, Centre for Cognitive Neuroscience, Member of ERN EpiCARE, Salzburg, Austria

^9^Neuroscience Institute, Christian-Doppler University Hospital, Paracelsus Medical University, Centre for Cognitive Neuroscience, Salzburg, Austria

^10^Institute of Public Health, Medical Decision-Making and HTA, UMIT – Private University for Health Sciences, Medical Informatics and Technology, Hall in Tyrol, Austria

**Corresponding author:** Eugen Trinka, MD, MSc, FRCP, Department of Neurology, Neurocritical Care and Neurorehabilitation, Christian Doppler University Hospital, Paracelsus Medical University Salzburg, Member of EpiCARE, Centre for Cognitive Neuroscience Salzburg, Ignaz Harrerstrasse 79, A-5020 Salzburg, Austria

Email: [eugen@trinka.at](mailto:eugen@trinka.at); ORCID iD: 0000-0002-5950-2692

## Table S1.

Congresses/meetings included in the grey literature search (July 2007 – September 2023)

| American Epilepsy Society Congress |
| --- |
| European Epilepsy Congress |
| European Paediatric Neurology Society Congress |
| International Congress of Neuropathology |
| International Epilepsy Congress |
| London-Innsbruck Colloquium on Status Epilepticus and Acute Seizures |
| World Congress of Neurology |

## Table S2.

Clinical evidence for the effectiveness of stiripentol in reducing emergency department admission and/or hospitalizations and rescue medication use in patients with epilepsy syndromes.

| **Reference** | **No. of pts** | **Diagnosis** | **ED visit or hospital admission** | **Rescue medication use** |
| --- | --- | --- | --- | --- |
| Alhakeem et al. (2018)^1^ | 3 | *SLC13A5*-related epileptic encephalopathy and history of SE leading to ED visits/hospital admissions >6 times/y | None in 3/3 pts | NR |
| Balestrini & Sisodiya (2017)^2^ | 13 | DS (8 with episodes of SE) | None in 1/13 pts | NR |
| Chiron et al. (2018)^3^ | 40 | DS | NR | ^1^V1: NR V2: Rescue benzodiazepines in 28% of pts V3: NR |
| Chiron et al. (2023)^4^ | 131 | DS | **None:** ^2^ *B/line,* 8.9% *V1,* 57.4%  *V2,* 87.8%  **≤1/mo:** ^2^ B/line, 33.3% *V1,* 4.3% *V2,* 0% **>1/mo:** ^2^ B*/line,* 57.8% *V1,* 38.3% *V2,* 12.2% | NR |
| Wirrell et al. (2013)^5^ | 82 | DS History of SE (55% of pts) | *Reduction in frequency:*^3^  STP, n=1/1 (100%)  STP+CLB, n=12/12 (100%) STP+VPA, n=3/5 (60%) STP+CLB+VPA n=18/19 (95%) | *Reduction in frequency*:^3^  STP n=2/4 (50%) STP+CLB n=25/25 (100%) STP+VPA n=5/10 (50%) STP+CLB+VPA n=26/33 (79%) |

^1^ Chiron et al. 2018 reported on treatment during childhood, and efficacy and safety outcomes for the following three time points: last visit before 15 years’ of age (V1; n=33), last visit before adulthood (V2; n=40), and the last visit in adulthood (V3; n=40).^3^

^2^Data are percentage patients at each time point.^4^ Chiron et al. 2023 provided data from three follow up visits: at STP initiation (baseline), after <6 months’ therapy (here designated V1), and at the last visit before 7 years’ of age ( i.e., after long-term therapy, here designated V2).^4^

^3^Reduction in frequency of ED/hospital admission, or rescue medication use reported only for the subgroup of patients with ED/hospital admission, or rescue medication use at least quarterly at baseline.^5^

Abbreviations: b/line, baseline; CLB, clobazam; ED, emergency department; DS, Dravet syndrome; mo, month(s); NR, not reported; pts, patients; SE, status epilepticus; V, visit; VPA, valproate; y, year.

## Figure S1.

Percentage of patients experiencing an adverse event during preventive treatment with stiripentol for epilepsy syndromes.


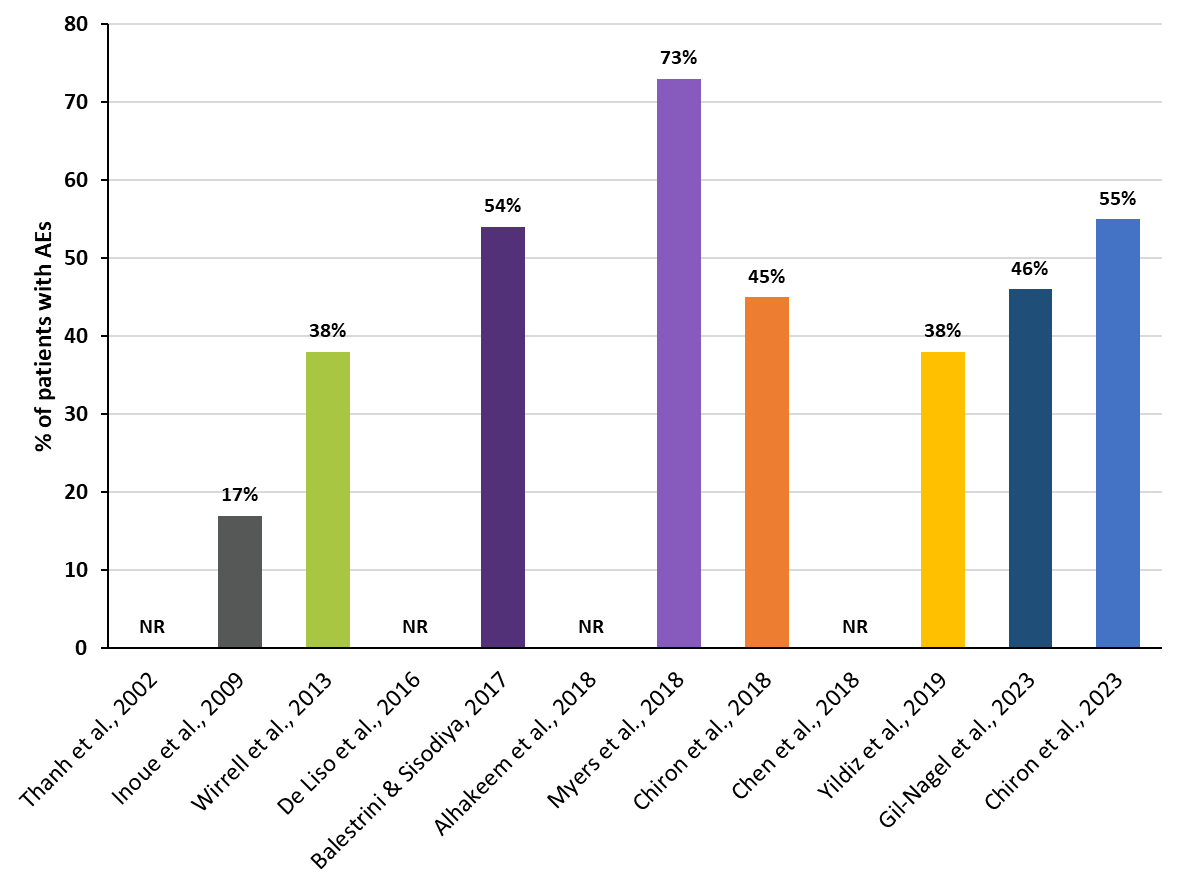


Data are the proportion of patients experiencing an AE. See Table 2 for details of each study (number of patients, etc.) Data from Inoue et al. (2009) are reported for an analysis from the ‘late period’,^6^ and for Chiron et al. (2018) are reported from visit 3, conducted when patients were aged a median of 18 years.^3^

AE, adverse event; NR, not reported.

## REFERENCES

1. Alhakeem A, Alshibani F, Tabarki B. Extending the use of stiripentol to SLC13A5-related epileptic encephalopathy. Brain Dev 2018;40:827-829.

2. Balestrini S, Sisodiya SM. Audit of use of stiripentol in adults with Dravet syndrome. Acta Neurol Scand 2017;135:73-79.

3. Chiron C, Helias M, Kaminska A, Laroche C, de Toffol B, Dulac O, et al. Do children with Dravet syndrome continue to benefit from stiripentol for long through adulthood? Epilepsia 2018;59:1705-1717.

4. Chiron C, Chemaly N, Chancharme L, Nabbout R. Initiating stiripentol before 2 years of age in patients with Dravet syndrome is safe and beneficial against status epilepticus. Dev Med Child Neurol 2023.

5. Wirrell EC, Laux L, Franz DN, Sullivan J, Saneto RP, Morse RP, et al. Stiripentol in Dravet syndrome: results of a retrospective U.S. study. Epilepsia 2013;54:1595-1604.

6. Inoue Y, Ohtsuka Y, Oguni H, Tohyama J, Baba H, Fukushima K, et al. Stiripentol open study in Japanese patients with Dravet syndrome. Epilepsia 2009;50:p. 197.
